# Author Correction: The structural dynamics of macropinosome formation and PI3-kinase-mediated sealing revealed by lattice light sheet microscopy

**DOI:** 10.1038/s41467-021-27411-4

**Published:** 2021-12-08

**Authors:** Shayne E. Quinn, Lu Huang, Jason G. Kerkvliet, Joel A. Swanson, Steve Smith, Adam D. Hoppe, Robert B. Anderson, Natalie W. Thiex, Brandon L. Scott

**Affiliations:** 1 Nanoscience and Nanoengineering, South Dakota School of Mines and Technology (South Dakota Mines), Rapid City, SD USA; 2 BioSNTR, South Dakota Mines, Rapid City, SD USA; 3 Department of Biology and Microbiology, South Dakota State University (SDSU), Brookings, SD USA; 4 BioSNTR, SDSU, Brookings, SD USA; 5 Department of Chemistry and Biochemistry, SDSU, Brookings, SD USA; 6 Department of Microbiology and Immunology, University of Michigan, Ann Arbor, MI USA

**Keywords:** 3-D reconstruction, Endocytosis, Cellular imaging, Image processing, Phagocytes

Correction to: *Nature Communications* 10.1038/s41467-021-25187-1, published online 10 August 2021.

In this article Robert B. Anderson should have been denoted as a corresponding author. This has now been corrected in the HTML and PDF version of this article.

