# Supplementary material for: The structural dynamics of macropinosome formation and PI3-kinase-mediated sealing revealed by lattice light sheet microscopy
Source: Nat Commun. 2021 Aug 10;12:4838. doi: 10.1038/s41467-021-25187-1 (PMC8355319; doi:10.1038/s41467-021-25187-1)
Supplement: Supplementary file 1 — Supplementary Information [file 41467_2021_25187_MOESM1_ESM.pdf]

Supplementary Information

The structural dynamics of macropinosome formation and PI3-kinase-mediated sealing revealed by lattice light sheet microscopy

Shayne E. Quinn[1,2], Lu Huang[3,4], Jason G. Kerkvliet[4,5], Joel A. Swanson[6], Steve Smith[1,2], Adam D. Hoppe[4,5], Robert B. Anderson[1,2], Natalie W. Thiex[3,4]* Brandon L. Scott[1,2]*

1 South Dakota School of Mines and Technology (South Dakota Mines), Nanoscience and Nanoengineering, Rapid City, SD. 2 BioSNTR, South Dakota Mines, Rapid City, SD. 3 South Dakota State University (SDSU), Department of Biology and Microbiology, Brookings, SD. 4 BioSNTR, SDSU, Brookings, SD. 5 SDSU, Department of Chemistry and Biochemistry, Brookings, SD.6 University of Michigan, Department of Microbiology and Immunology, Ann Arbor, MI.

* Co-corresponding authors: natalie.thiex@sdstate.edu, brandon.scott@sdsmt.edu

Supplementary Figures

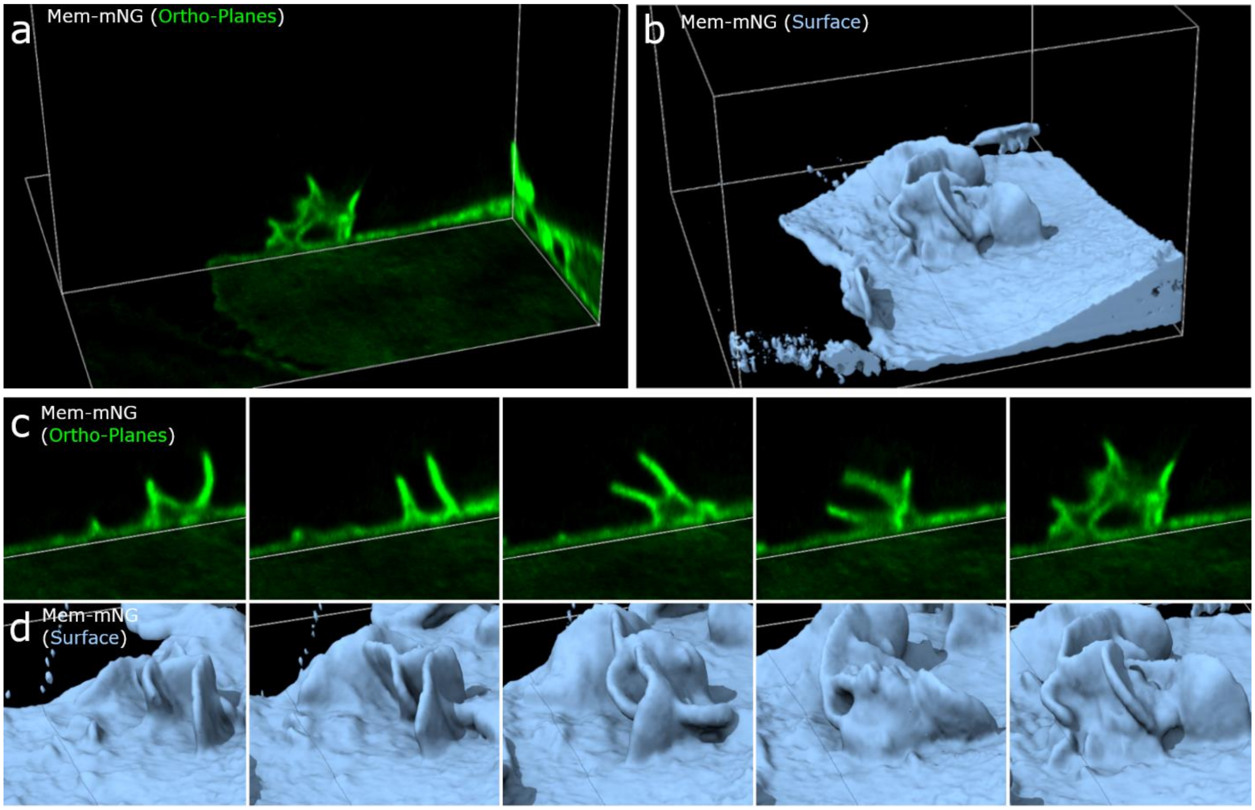

Supplementary Figure 1. Constitutive macropinocytosis and the importance of reducing the dimensionality of data. a) Orthoplane (left) and isosurface (right) views of Mem-mNG show the subsurface macropinosome and the complex structure of the full surface (Region 20x20x15 µm). b) Orthoplane montage of Mem-mNG depicting constitutive planar view of macropinocytosis where two ruffles extend from the cell membrane, circularize, and connect to form a macropinosome. c) Close view of the respective orthoplane and isosurface views of Mem-mNG showing the complex structures and the multiple membrane ruffles involved in the macropinocytic event.

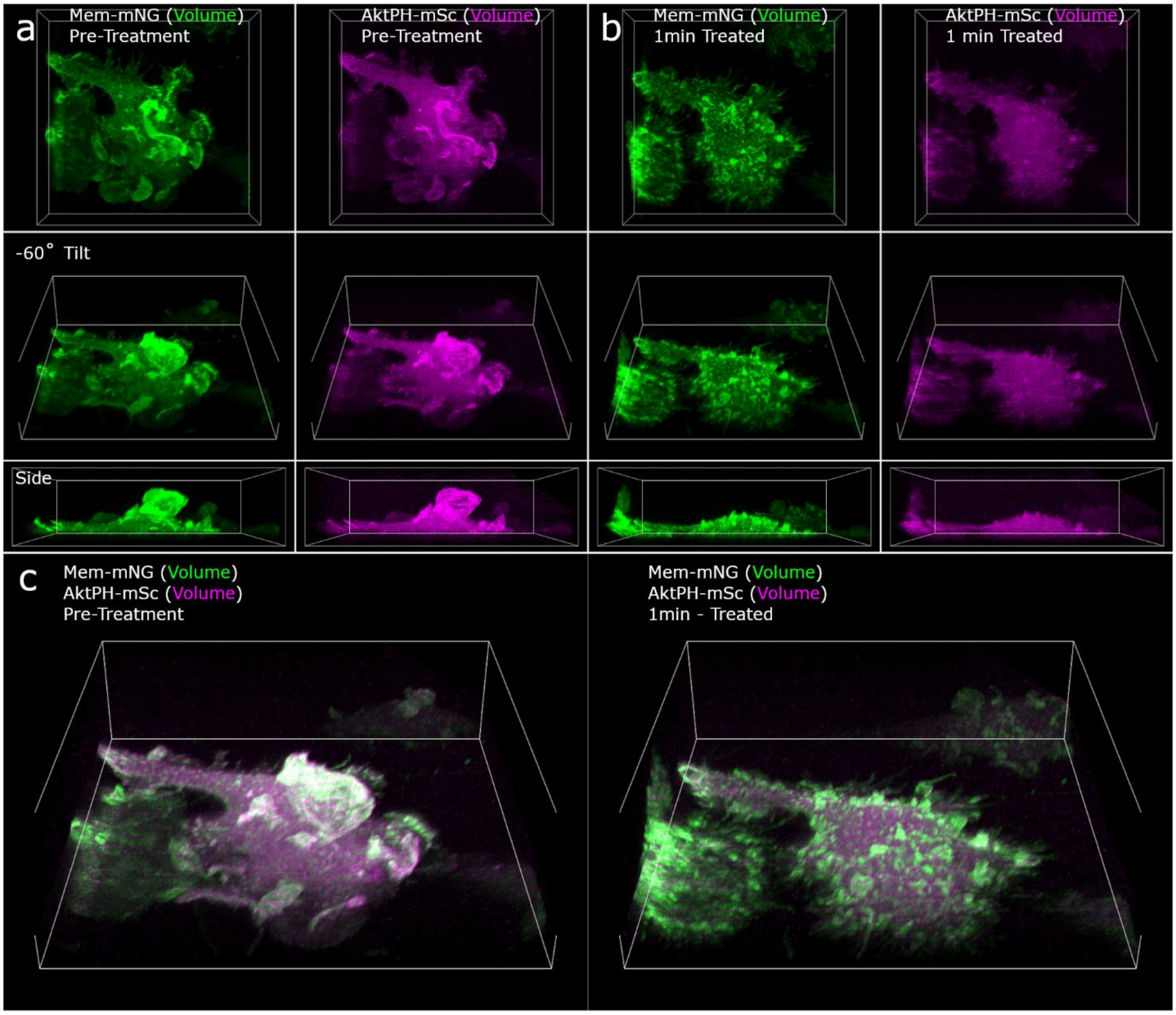

Supplementary Figure 2. LY294002 drug treatment response. a) Two columns showing different angles of a split volume representations for Mem-mNG and AktPH-mSc of an untreated macrophage actively ruffling and creating macropinosomes (Region 71x69x20 µm). b) Two columns showing the same cell treated with LY294002 displayed as split volumes of Mem-mNG and AktPH-mSc one minute after drug treatment (Region 71x69x20 µm). c) Composite volume representations of pre-treatment (left) and one minute post LY294002 drug treatment (right) (Region 71x69x20 µm).

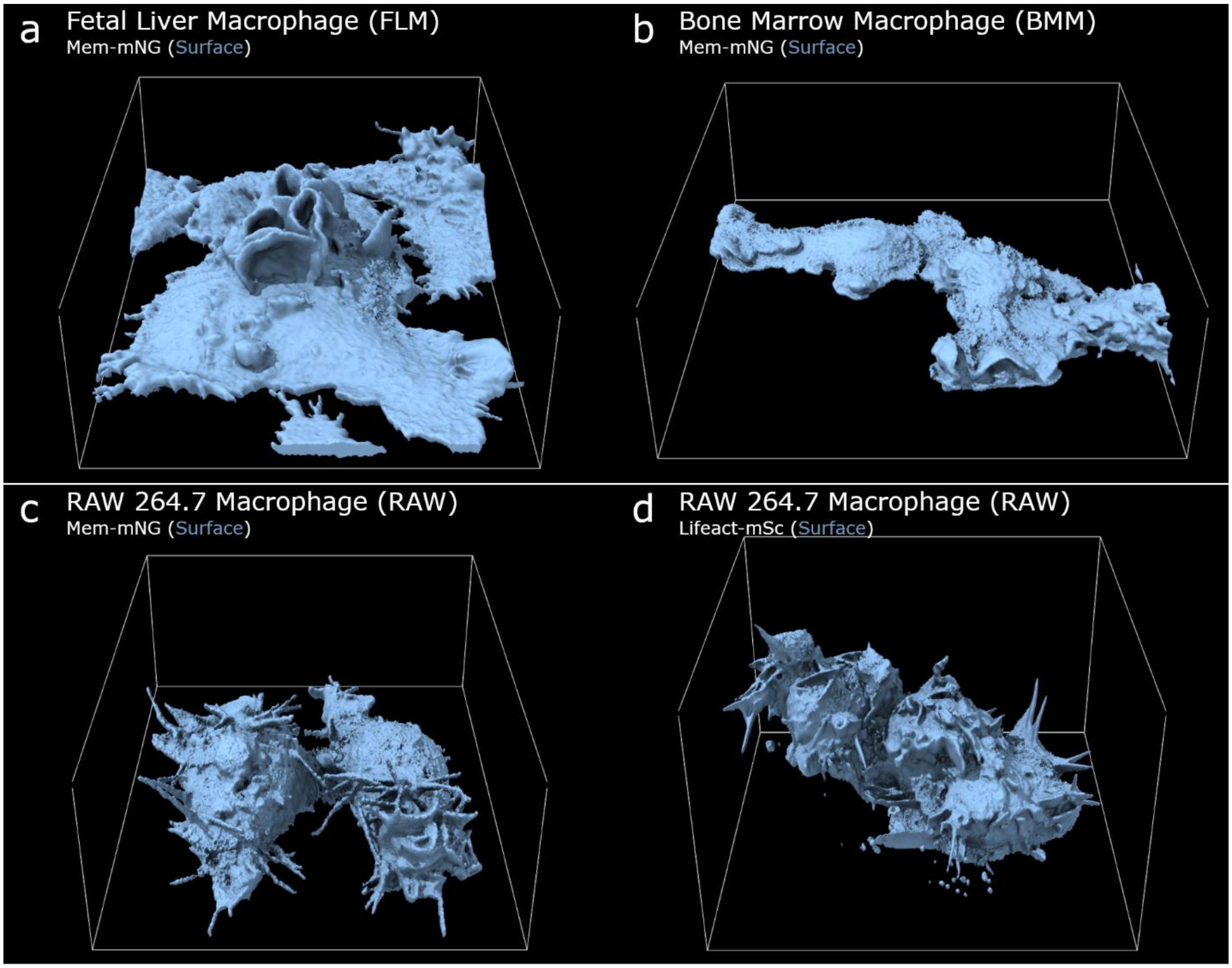

Supplementary Figure 3. Cell line variability and cross comparison. Isosurface view of Mem-mNG showing the general membrane structure observed by LLSM: a) fetal liver macrophage (Region 47x59x19 µm), b) bone marrow derived macrophage (Region 71x68x22 µm), and c) RAW264.7 (Region 50x38x17 µm). d) Isosurface view of Lifeact-mSc expressed in RAW264.7 demonstrating a similar morphology with filopodial extensions (Region 49x49x30 µm).
