# Supplementary material for: The structural dynamics of macropinosome formation and PI3-kinase-mediated sealing revealed by lattice light sheet microscopy
Source: Nat Commun. 2021 Aug 10;12:4838. doi: 10.1038/s41467-021-25187-1 (PMC8355319; doi:10.1038/s41467-021-25187-1)
Supplement: Supplementary file 19 — Description of Additional Supplementary Information [file 41467_2021_25187_MOESM19_ESM.pdf]

Description of Additional Supplementary Files

File Name: Supplementary Movie 1

Description: Corresponds to Fig. 1a, c. Isosurface and volumetric intensity renderings (Green Mem-mNG; Magenta AktPH-mSc) provides different methods of visualizing the formation (surface) and trafficking of macropinosomes and AktPH-mSc accumulation (volume). Movie timestamps highlight the following events: AktPH enriched ruffle development (02:42), newly formed macropinosomes (06:29; 08:51; 10:58), and post closure AktPH recruitment (11:19). Frame rate of ~7s and each region is 68x72x25 µm.

File Name: Supplementary Movie 2

Description: Corresponds to Fig. 1d. Isosurface rendering in conjunction with orthogonal planes moved through a volume provide ~0.1 µm thick planes to help visualize the internal and surface activity of Mem-mNG and AktPH-mSc including macropinosome closures (02:41; 05:15), membrane rich ruffles (00:00; 02:34; 05:08), previously formed internal macropinosomes (05:15 during scan), and mSc-Akt localization around a closed macropinosome (05:15 during scan). Framerate of ~7s and two subregions each 29x30x19 µm.

File Name: Supplementary Movie 3

Description: Corresponds to Fig. 2a. Isosurface and dual-volumetric intensity projection of Mem-mNG and AktPH-mSc with a 25-degree tilt shows a variety of formation events. Several early formations occur prior to relaxation of the plasma membrane (01:58), followed by the development of another AktPH rich ruffle (02:48), membrane closure into a macropinosome (03:07 -> 3:13), and finally post closure recruitment of AktPH (03:25). Several macropinosomes form that are indicated by the recruitment of AktPH post closure (3:57; 07:16) and subsequently trafficked toward one another to merge (4:53; 05:18; 05:30; 07:54). Frame rate of ~6.25s and region of 21x19x15 µm.

File Name: Supplementary Movie 4

Description: Corresponds to Fig. 2c. Mesh rendering of Mem-mNG and volumetric intensity projection of AktPH-mSc (Magenta-Hot) using a 90-degree tilt. The second play through contains a pause to emphasize the frame shown in Fig 2c and highlight the AktPH rich membrane ruffles (02:48) and the post closure recruitments (03:25; 03:57). Frame rate ~6.25s region of 21x19x15 µm.

File Name: Supplementary Movie 5

Description: Corresponds to Fig. 3a, b. Isosurface in conjunction with three volumetric intensity renderings (Green Mem-mNG; Magenta AktPH-mSc) display a traditional formation in an untreated cell including the initial ruffle (00:00) that vertically extends and begins to form a tidal wave (01:31) back toward the surface of the cell, with membrane scission (03:24 -> 03:31) and finally the post closure recruitment for the largest macropinosome (08:07). Frame rate of 7s and Region of 12x13x10 µm.

File Name: Supplementary Movie 6

Description: Corresponds to Fig. 3d, e. Isosurface with three volumetric intensity renderings (Green Mem-mNG; Magenta AktPH-mSc) on an LY294002 treated macrophage showing the initial ruffle with uniform AktPH throughout the cytosol and ruffle (00:00), attempted closure of the ruffle (00:30), continued compression of the attempted macropinosome (00:30 -> 02:27), becoming un-trackable within the cytosol with no AktPH recruitment to the attempted macropinosome. (Tracking done manually using orthogonal planes) Frame rate of 6.15s and Region 10x12x10 µm.

File Name: Supplementary Movie 7

Description: Corresponds to Supplementary Figure 2 Two color volumetric intensity renderings (Green Mem-mNG; Magenta AktPH-mSc) showing a pre-treated macrophage creating large dorsal ruffles rich with PI3K activity. After ~9 minutes of imaging, the cells were introduced to LY294002 and the same cell was reimaged showing a rapid reduction in ruffling and PI3K activity. After ~9 minutes of treatment, we show a second cell showing the start of returning ruffle activity on the same coverslip. Finally, a third cell was imaged starting at ~18 minutes into drug treatment showing the return of moderate ruffle formations but no increased PI3K activity. Frame rate of 7s and region 71x69x20 µm.

File Name: Supplementary Movie 8

Description: Corresponds to Fig. 4a, b. Isosurface alongside three volumetric intensity renderings (Green Mem-mNG; Magenta AktPH-mSc) shows the formation of macropinosomes at the base of a larger ruffle. Membrane relaxes (00:56), small protrusions form with increased AktPH (01:17), two small ruffles, one in the back and one in the front mergers with the larger ruffle (01:32 -> 01:39) followed by post closure AktPH recruitment (01:53). Frame rate of 7s and region of 11x9x12 µm.

File Name: Supplementary Movie 9

Description: Corresponds to Fig. 5c. Isosurface and dual-volumetric intensity projection of Mem-mNG and AktPH-mSc showing a smooth and relaxed membrane (00:00). A single macropinosome forms (05:06) followed by a large increased in membrane activity (06:35) resulting in a significant number of macropinosomes, indicated by the post closure AktPH recruitment, that turns into the chaotic membrane structure (06:35 -> 13:18). Frame rate of 8s and region size of 27x22x16 µm.

File Name: Supplementary Movie 10

Description: Corresponds to Fig. 5e. Side view of Mem-mNG isosurface and mesh membrane with volumetric AktPH (Magenta Hot) shows the continued AktPH localization within the extending membrane structure (06:35). Utilizing the Magenta-Hot LUT regions displaying in white represent the increase in AktPH post macropinosome closure signifying a formed macropinosome (05:55; 06:59; 08:12; 11:50). Frame rate of 8s and region size of 27x22x16 µm.

File Name: Supplementary Movie 11

Description: Corresponds to Fig. 6a, b, c. CSF-1 starved macrophage displayed using Mem-mNG isosurface/mesh/volume and AktPH volumes as magenta-hot under the mesh and Magenta alongside the volume membrane. The macrophage was imaged 07:41 prior to stimulation and reimaged one minute after CSF-1 stimulation (08:41) providing time to ensure instrument and imaging conditions had not changed. The starved cell ruffled and formed macropinosomes similar to the conventional cells (00:06; 01:46; 03:26) and upon stimulation (08:41) a large circular dorsal ruffle forms corralling the AktPH to one concentrated spot in the cell (16:22). Frame rate of 6.25s and region size of 49x60 µm.

File Name: Supplementary Movie 12

Description: Corresponds to Fig. 6d. The brightfield view starts promptly after stimulation showing the majority of macrophages performing the similar dorsal membrane clearing seen in the LLSM imaging.

File Name: Supplementary Movie 13

Description: Corresponds to Fig. 7a, b. LPS Stimulation. Isosurface of Mem-mNG and dual-color volumetric intensity projections of Mem-mNG and AktPH-mSc showing the activity of an LPS treated cell. Initial imaging starts (00:00) with a cluster of membrane rich in AktPH that goes on to create many macropinosomes as it expands toward the upper right region of the field of view. The activity changes directions toward the upper left region of the cell and proceeds to move counterclockwise (01:14), over the nucleus and back to the initial location ending at (04:42). Additional macropinosomes are seen forming on the left region with the increased AktPH flare up post closure (04:42). Finally, several formations occur in the bottom right of the cell (05:05 – 08:18) many of which go on to merge with one another. Framerate of ~7s and Region of 68x77x21 µm.

File Name: Supplementary Movie 14

Description: Corresponds to Fig. 7c, d. Non-treated control. Isosurface of Mem-mNG and dual-color volumetric intensity projections of Mem-mNG and AktPH-mSc showing the imaging of a nontreated cell (10:47). Two AktPH rich regions of membrane ruffling are seen in the bottom right of the cell (02:17) that form several small macropinosomes, indicated by a spike in AktPH around the formed macropinosome. The cell shows activity that is representative of the untreated experiments including macropinosome formations, exploration, and overall membrane ruffling. Framerate of ~6.5s and Region of 68x77x21 µm.

File Name: Supplementary Movie 15

Description: Corresponds to Supplementary Figure 3b. Isosurface rendering of Mem-mNG in a BMM. The cell shows activity that is representative of the BMM population including macropinosome formations, surface spreading, and overall membrane ruffling. Framerate of ~4.2s and region 71x68x22 µm.

File Name: Supplementary Movie 16

Description: Corresponds to Supplementary Figure 3c. Isosurface rendering of Mem-mNG in an RAW264.7 cell. The cell shows activity that is representative of the RAW264.7 population including tentpole extensions, rounded shape, and general membrane activity. Framerate of ~3.5s and region 50x38x17 µm.
