# Supplementary material for: The structural dynamics of macropinosome formation and PI3-kinase-mediated sealing revealed by lattice light sheet microscopy
Source: Nat Commun. 2021 Aug 10;12:4838. doi: 10.1038/s41467-021-25187-1 (PMC8355319; doi:10.1038/s41467-021-25187-1)
Supplement: Supplementary file 20 — Reporting Summary [file 41467_2021_25187_MOESM20_ESM.pdf]

Corresponding author(s): Natalie W. Thiex
Brandon L. Scott

Last updated by author(s): 07/05/2021

# Reporting Summary

Nature Research wishes to improve the reproducibility of the work that we publish. This form provides structure for consistency and transparency in reporting. For further information on Nature Research policies, see our Editorial Policies and the Editorial Policy Checklist.

## Statistics

For all statistical analyses, confirm that the following items are present in the figure legend, table legend, main text, or Methods section.

| n/a | Confirmed | |
|---|---|---|
| ☐ | ☒ | The exact sample size ($n$) for each experimental group/condition, given as a discrete number and unit of measurement |
| ☐ | ☒ | A statement on whether measurements were taken from distinct samples or whether the same sample was measured repeatedly |
| ☒ | ☐ | The statistical test(s) used AND whether they are one- or two-sided<br>*Only common tests should be described solely by name; describe more complex techniques in the Methods section.* |
| ☒ | ☐ | A description of all covariates tested |
| ☒ | ☐ | A description of any assumptions or corrections, such as tests of normality and adjustment for multiple comparisons |
| ☐ | ☒ | A full description of the statistical parameters including central tendency (e.g. means) or other basic estimates (e.g. regression coefficient) AND variation (e.g. standard deviation) or associated estimates of uncertainty (e.g. confidence intervals) |
| ☒ | ☐ | For null hypothesis testing, the test statistic (e.g. $F$, $t$, $r$) with confidence intervals, effect sizes, degrees of freedom and $P$ value noted<br>*Give P values as exact values whenever suitable.* |
| ☒ | ☐ | For Bayesian analysis, information on the choice of priors and Markov chain Monte Carlo settings |
| ☒ | ☐ | For hierarchical and complex designs, identification of the appropriate level for tests and full reporting of outcomes |
| ☒ | ☐ | Estimates of effect sizes (e.g. Cohen's $d$, Pearson's $r$), indicating how they were calculated |

*Our web collection on statistics for biologists contains articles on many of the points above.*

## Software and code

Policy information about availability of computer code

| | |
|---|---|
| Data collection | Microscope control and acquisition used LabView: v4.03952.0012 |
| Data analysis | LLSpy: v0.4.8 was used to process the microscopy data. ChimeraX: v1.1 was used to generate the volumetric renderings. |

For manuscripts utilizing custom algorithms or software that are central to the research but not yet described in published literature, software must be made available to editors and reviewers. We strongly encourage code deposition in a community repository (e.g. GitHub). See the Nature Research guidelines for submitting code & software for further information.

## Data

Policy information about availability of data

All manuscripts must include a data availability statement. This statement should provide the following information, where applicable:
- Accession codes, unique identifiers, or web links for publicly available datasets
- A list of figures that have associated raw data
- A description of any restrictions on data availability

The processed LLSM data along with one version of raw data are available through the Cell Image Library (CIL) database under accession codes CIL: 54592-54604 available at http://cellimagelibrary.org/groups/54592. Additional raw data are available upon request.

# Field-specific reporting

Please select the one below that is the best fit for your research. If you are not sure, read the appropriate sections before making your selection.

☒ Life sciences ☐ Behavioural & social sciences ☐ Ecological, evolutionary & environmental sciences

For a reference copy of the document with all sections, see nature.com/documents/nr-reporting-summary-flat.pdf

# Life sciences study design

All studies must disclose on these points even when the disclosure is negative.

| | |
|---|---|
| Sample size | No sample size calculation was performed. The experiments were repeated at least three times resulting in approximately 100 macropinocytic events for analysis. This is consistent with established norms in the field. |
| Data exclusions | Out of focus movies, cells that were only partially in the field of view during the movie and clearly dead/dying cells were excluded. |
| Replication | Each experiment was performed in triplicate over multiple days. All attempts at replication were successful. |
| Randomization | Treatment and control groups were plated in the same well prior to being moved to separate wells for stimulation when needed and all in-focus and in field of view movies were used for analysis. |
| Blinding | The experiments and analysis were not blinded as representative cells on the coverslip were found and position marked prior to any external stimulation. All movies with a cell residing in the field of view and in focus were used for analysis. |

# Reporting for specific materials, systems and methods

We require information from authors about some types of materials, experimental systems and methods used in many studies. Here, indicate whether each material, system or method listed is relevant to your study. If you are not sure if a list item applies to your research, read the appropriate section before selecting a response.

## Materials & experimental systems

| n/a | Involved in the study |
|---|---|
| ☒ | ☐ Antibodies |
| ☐ | ☒ Eukaryotic cell lines |
| ☒ | ☐ Palaeontology and archaeology |
| ☒ | ☐ Animals and other organisms |
| ☒ | ☐ Human research participants |
| ☒ | ☐ Clinical data |
| ☒ | ☐ Dual use research of concern |

## Methods

| n/a | Involved in the study |
|---|---|
| ☒ | ☐ ChIP-seq |
| ☒ | ☐ Flow cytometry |
| ☒ | ☐ MRI-based neuroimaging |

## Eukaryotic cell lines

Policy information about cell lines

| | |
|---|---|
| Cell line source(s) | FLMs were derived from gestational day 15-19 mouse fetuses (Male and Female) from C57BL/6J Female mice. NIH293T and Raw 264.7 were obtained from ATCC. Bone marrow derived macrophages were obtained from C57BL/6J mice. |
| Authentication | None of the cell lines were authenticated. |
| Mycoplasma contamination | Cells were cultured in the presence of Plasmocin™ - Prophylactic from InvivoGen, and were routinely tested using PlasmoTest™ - Mycoplasma Detection Kit from InvivoGen to ensure no mycoplasma contamination occurred. |
| Commonly misidentified lines (See ICLAC register) | No commonly misidentified lines were used. |
